# Metagenomic sequencing reveals altered gut microbial compositions and gene functions in patients with non-segmental vitiligo

**DOI:** 10.1186/s12866-023-03020-7

**Published:** 2023-09-22

**Authors:** Mei Luan, Mengtian Niu, Pengju Yang, Dan Han, Yudan Zhang, Weizhe Li, Qiannan He, Yixin Zhao, Binyue Mao, Jianan Chen, Kuanhou Mou, Pan Li

**Affiliations:** 1 Department of Dermatology, the Frist Affiliated Hospital of Xi’an Jiaotong University, 277 West Yanta Road, 710061 Xi’an, Shaanxi People’s Republic of China; 2 Center for Translational Medicine, the Frist Affiliated Hospital of Xi’an Jiaotong University, 277 West Yanta Road, 710061 Xi’an, Shaanxi People’s Republic of China

**Keywords:** Vitiligo, Gut microbiota, Metagenomic sequencing

## Abstract

**Background:**

Vitiligo has been correlated with an abnormal gut microbiota. We aimed to systematically identify characteristics of the gut microbial compositions, genetic functions, and potential metabolic features in patients with non-segmental vitiligo.

**Methods:**

Twenty-five patients with non-segmental vitiligo and 25 matched healthy controls (HCs) were enrolled. Metagenomic sequencing and bioinformatic analysis were performed to determine the gut microbiota profiles. Differences in gut microbiota diversity and composition between patients with vitiligo and HCs were analyzed. Gene functions and gut metabolic modules were predicted with the Kyoto Encyclopedia of Gene and Genomes (KEGG) and MetaCyc databases.

**Results:**

Compared with HCs, alpha diversity of intestinal microbiome in vitiligo patients was significantly reduced. At the species level, the relative abundance of *Staphylococcus thermophiles* was decreased, and that of *Bacteroides fragilis* was increased in patients with vitiligo compared with those of the HCs. Linear discriminant analysis (LDA) effect size (LEfSe) analysis revealed representative microbial markers of *Lachnospiraceae_bacterium_BX3*, *Massilioclostridium_coli*, *TM7_phylum_sp_oral_taxon_348* and *Bacteroides_fragilis* for patients with vitiligo. KEGG gene function analysis showed that the NOD-like receptor signaling pathway was significantly enriched in patients with vitiligo. Gut metabolic modules (GMMs) analysis showed that cysteine degradation was significantly down-regulated, and galactose degradation was up-regulated in patients with vitiligo. A panel of 28 microbial features was constructed to distinguish patients with vitiligo from HCs.

**Conclusions:**

The gut microbial profiles and genetic functions of patients with vitiligo were distinct from those of the HCs. The identified gut microbial markers may potentially be used for earlier diagnosis and treatment targets.

**Supplementary Information:**

The online version contains supplementary material available at 10.1186/s12866-023-03020-7.

## Background

Vitiligo is an acquired chronic pigmentary disorder involving loss of cutaneous melanocytes. The incidence rate of vitiligo is about 0.5–2% worldwide [1]. Vitiligo is considered a CD8 + T-cell-mediated autoimmune disease. Perilesional skin is highly enriched in auto-reactive CD8 + T cells, which are activated by melanocyte-specific antigen and play a key role in the destruction of melanocytes [2–4].

Disruption of gut microbes affects the whole body's immune system, the gut microbiome has been involved in various autoimmune disease such as systemic lupus erythematosus [5], rheumatoid arthritis [6], and inflammatory bowel disease [7]. Studies demonstrate that the gut microbiome’s influence extends beyond the intestine to distant organs such as the brain, liver, and lung [8–10]. Recent researches in gut microbiome and dermatosis have validated the concept of the gut-skin axis [11]. Metagenomic sequencing of fecal samples showed differences in the composition and function of the gut microbiota between psoriasis patients and HCs, supporting a potential link between gut microbiota and psoriasis [12]. A study found that gut-derived *Bifidobacterium* mediated tryptophan metabolism to attenuate atopic dermatitis via the gut-skin axis; thus, microbial metabolites from tryptophan may be the means by which *Bifidobacteria* alleviate atopic dermatitis via the aryl hydrocarbon receptor signaling pathway [13].

Evidence has shown that an altered gut microbiome may contribute to the vitiligo pathogenesis. Hanene et al. compared vitiligo patients with HCs and found gut dysbiosis via reduced richness and bacterial species distributions in patients with vitiligo [14]. A case-control study of 30 patients with vitiligo found increased microbial diversity with dominant contributions of *Corynebacterium* 1 and *Psychrobacter* in vitiligo [15]. Dellacecca et al. studied a mouse model of vitiligo and found that oral ampicillin accelerated the onset and progression of skin and hair depigmentation, indicating that alteration of specific intestinal bacterial communities affects vitiligo disease activity [16]. From these studies, we hypothesized that the etiology and pathophysiologic mechanisms of vitiligo may be correlated with dysfunction of the gut-skin axis; thus, the composition and functional capabilities of the intestinal flora associated with vitiligo require systematic examination. Previous studies have been based on 16s rRNA sequencing, whereas we used metagenomic shotgun sequencing, which is characterized by high-throughput analysis and complete sequencing information, to identify more low-abundance microbial communities. Metagenomic sequencing enables better microbiota characterization and can more accurately predict microbial genetic functions and potential metabolic features [17, 18]. To date, metagenomic sequencing has not been used to study the role of gut microbes in vitiligo.

In this study, we used metagenomic sequencing to analyze species-level gut microbiota composition and potential functional and metabolic characteristics between vitiligo and HCs. The results will help determine the pathogenesis and potential treatments for vitiligo.

## Results

### Participant demographics

Fifty stool samples were collected: 25 from patients with advanced non-segmental vitiligo (18 males and 7 females, mean age: 31.23 ± 6.81 years) and 25 from matched HCs (18 males and 7 females, mean age: 30.47 ± 9.03 years). All patients were evaluated for Vitiligo Disease Activity (VIDA) scores (2.80 ± 1.08). Table 1 provides the detailed characteristics of study groups.

**Table 1:** Characteristics of study groups

Variables	Healthy Control	Patients with Vitiligo	*P*-value
Subjects (n)	25	25	-
Sex (male/female)	18/7	18/7	0.551*
Age, years, mean ± SD	31.23 ± 6.81	30.47 ± 9.03	0.627#
Body mass index (BMI), kg/m^2^, mean ± SD	23.66 ± 3.02	22.81 ± 4.05	0.181#
Smoking, n (%)	7 (28)	5 (20)	0.742*
Alcohol consumption, n (%)	9 (36)	6 (24)	0.538*
Diet n (%)			0.703*
High protein diets	6 (24)	5 (20)	
High carbohydrate diets	8 (32)	6 (24)	
High fiber diets	5 (20)	4 (16)	
Balanced dieters	6 (24)	10 (40)	
Vitiligo Disease Activity(VIDA) score, mean ± SD	-	2.80 ± 1.08	
Type of vitiligo	-	Non-segmental	
Activity of vitiligo	-	Advanced	
Disease Duration, month, mean ± SD	-	41.28 ± 50.28	
Family history (%)	-	4 (16%)	

^*^Fisher. test

^#^Wilcoxon. test

**Fig. 1:**
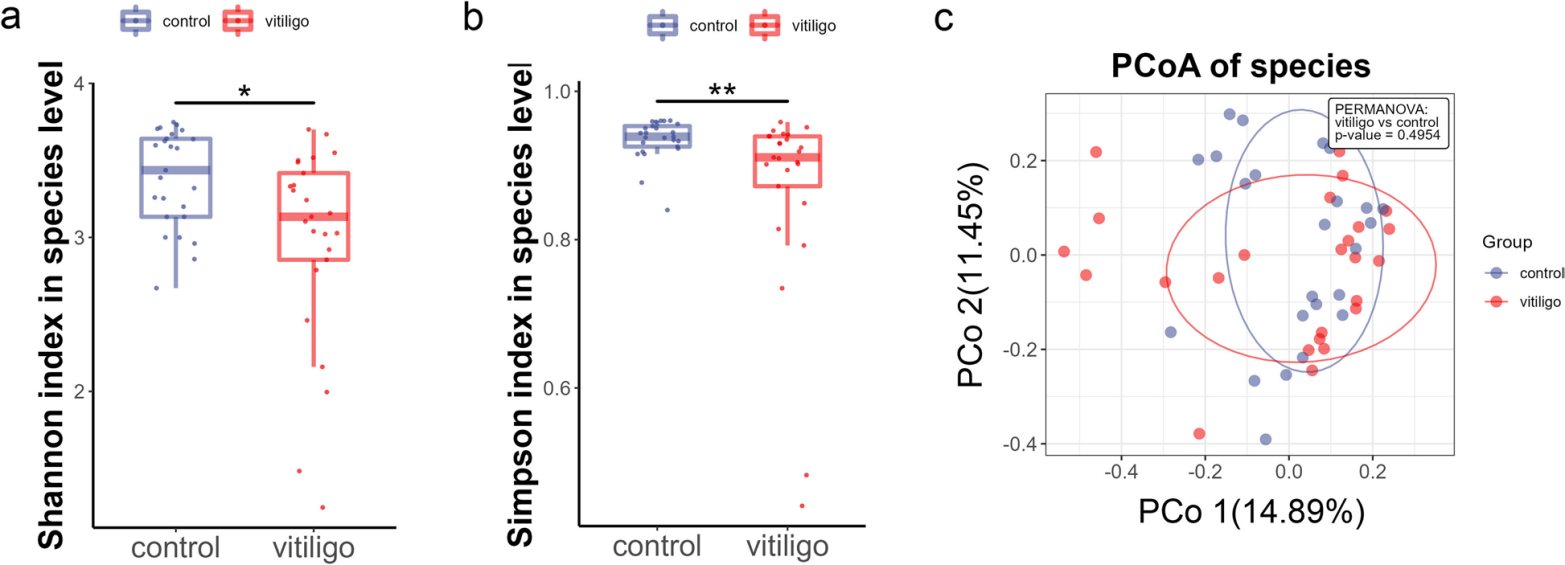
Gut microbial α-diversity was significantly reduced in vitiligo patients; β-diversity was not. **a**,** b** Decreased gut microbial α-diversity in vitiligo patients. Vitiligo patients had lower Shannon (**a**) and Simpson (**b**) indexes than did the HCs (Wilcoxon rank-sum test, *P* < 0.05, boxplots are shown as medians, 5%‒95% confidence interval). **c** PCoA plot representing β-diversity in vitiligo patients (red dots) and HCs (blue dots) (PEMANOVA test, *P* = 0.495).

### Gut microbial α-diversity was decreased in patients with vitiligo

First, we examined discrepancies of the gut microbiota diversity between vitiligo patients and HCs. α-diversity and β-diversity was measured to figure distinction between the groups. Shannon (Fig. 1a; Supplementary Table S1, Wilcoxon rank-sum test, *P =* 0.011) and Simpson (Fig. 1b; Supplementary Table S1, Wilcoxon rank-sum test, *P =* 0.002) indexes showed that the α-diversity of the gut microbiota was significantly lower in vitiligo patients than in HCs, whereas principal coordinate analysis (PCoA) showed that the β-diversity did not significantly differ (Fig. 1c; Supplementary Table S2, PEMANOVA test, *P* = 0.495).

**Fig. 2:**
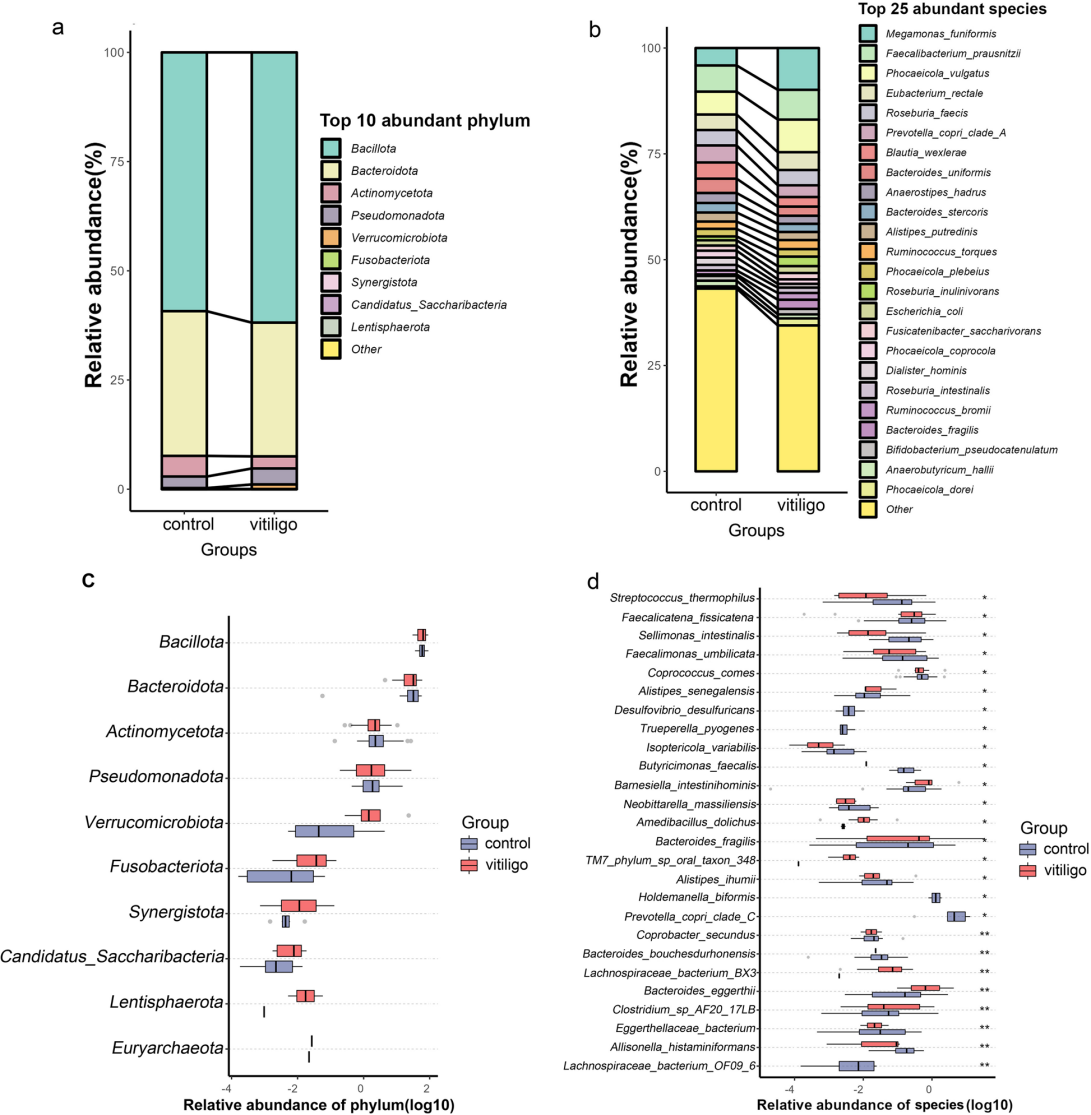
Gut microbiota compositions and relative abundances. **a**,** b** Composition and relative proportions of gut microbes between the two groups at the phylum (**a**: top 10 phyla) and species (**b**: top 25 species) levels. **c** Comparison of gut microbiotas between vitiligo patients and HCs at the phylum level. **d** 26 gut microbial species differed significantly in vitiligo patients compared with those of the HCs. Boxplots show relative abundances. Wilcoxon rank-sum test: asterisks denote *P* < 0.05; double asterisks means *P* < 0.01

### Gut microbial compositions were disturbed in patients with vitiligo

The top ten most abundant phyla included *Bacillota, Bacteroidota, Actinomycetota*, and *Pseudomonadota* et al. (Fig. 2a). In patients with vitiligo, *Bacillota* and *Bacteroidota* constituted 92.49% of the total microbial population. The relative abundance of *Bacillot*a to *Bacteroidota* ratio was similar in the vitiligo group and the control group (2.02%vs1.78%; *P* > 0.05). Additionally, in the phylum level, the relative abundance of *Bacillota* was higher (61.86%vs59.25%; *P* > 0.05) and the relative abundance of *Bacteroidota* was lower (30.62%vs33.12%; *P* > 0.05) in patients compared with HCs (Fig. 2a), although the differences were not significant (Fig. 2c). The top 25 most abundant species in patients with vitiligo included *Megamonas_funiformis*, *Faecalibacterium_prausnitzii*, *Phocaeicola_vulgatus*, *Eubacterium_rectale* and *Roseburia_faecis* et al. (Fig. 2b).

At the species level, 26 gut microbes differed significantly between the patients and HCs (*P* < 0.05; Fig. 2d; Supplementary Table S3). The abundances of 20 gut microbial species were lower in patients than in HCs. Patients with vitiligo had decreased abundances of *Staphylococcus thermophiles*, *Sellimonas intestinalis*, *Coprococcus comes*, *Clostridium_sp_AF20_17LB*, *Coprobacter secundus*, *Bacteroides_bouchesdurhonensis*, *Eggerthellaceae_bacterium*, and *Allisonella_histaminiformans*, and higher abundances of *Lachnospiraceae bacterium*, *TM7_phylum_sp_oral_taxon_348*, *Bacteroides_fragilis*, *Amedibacillus_dolichus*, and *Barnesiella intestinihominis* than did the HCs.

Then, correlation analysis was conducted between the identified differential bacteria species and the VIDA score, and it was found that there was no significant statistically difference between them (Supplementary Table S4).

**Fig. 3:**
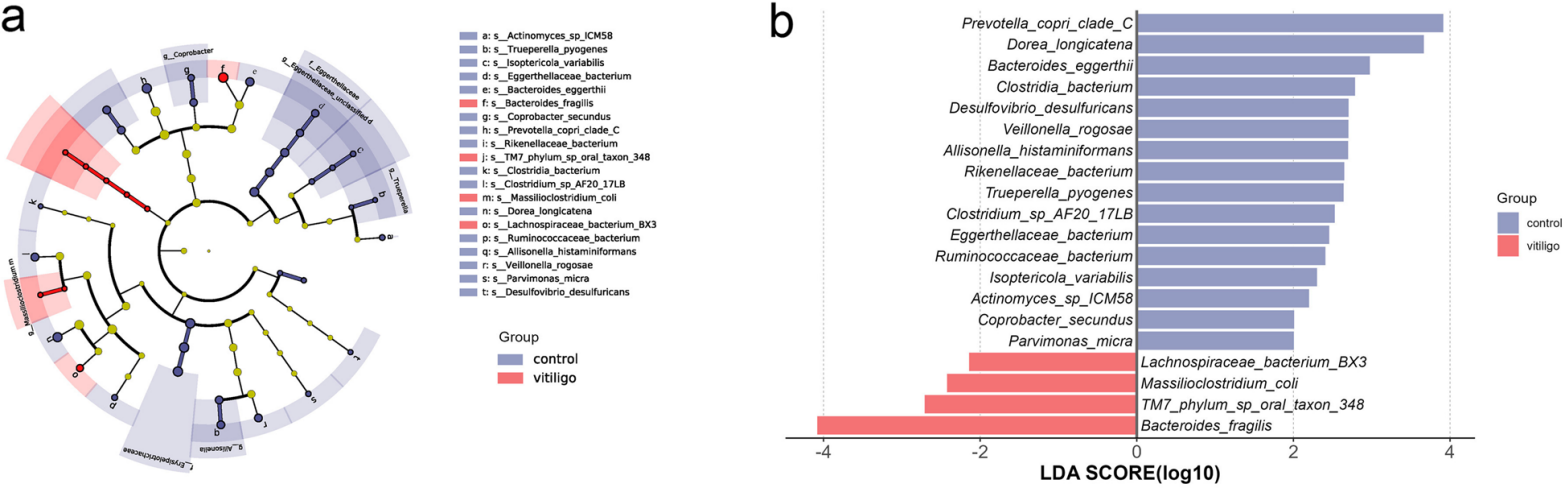
Gut microbial biomarkers correlated with vitiligo. **a** Cladogram generated via LEfSe analysis indicating the specific microbial species in the vitiligo patients and HCs. **b** LDA score histograms identify difference-rich bacteria between vitiligo patients and HCs (LDA score > 2.0)

### Representative gut microbial markers for vitiligo patients and HCs

We used LEfSe analysis to further investigate significantly different bacteria in patients with vitiligo and tested for statistical significance by combining standard tests with additional analyses that examined biological consistency and association of effects. Several species, including *Bacteroides_fragilis*, *TM7_phylum_sp_oral_taxon_348*, *Massilioclostridium_coli* and *Lachnospiraceae_bacterium_BX3* were significantly enriched in patients with vitiligo, whereas *Prevotella_copri_clade_C* and *Dorea_longicatena* were negatively associated with these patients (Fig. 3a, b). The identified taxa are highlighted on pedigree maps to indicate significant differences in phylogenetic distribution (Fig. 3b; Supplementary Table S5).

**Fig. 4:**
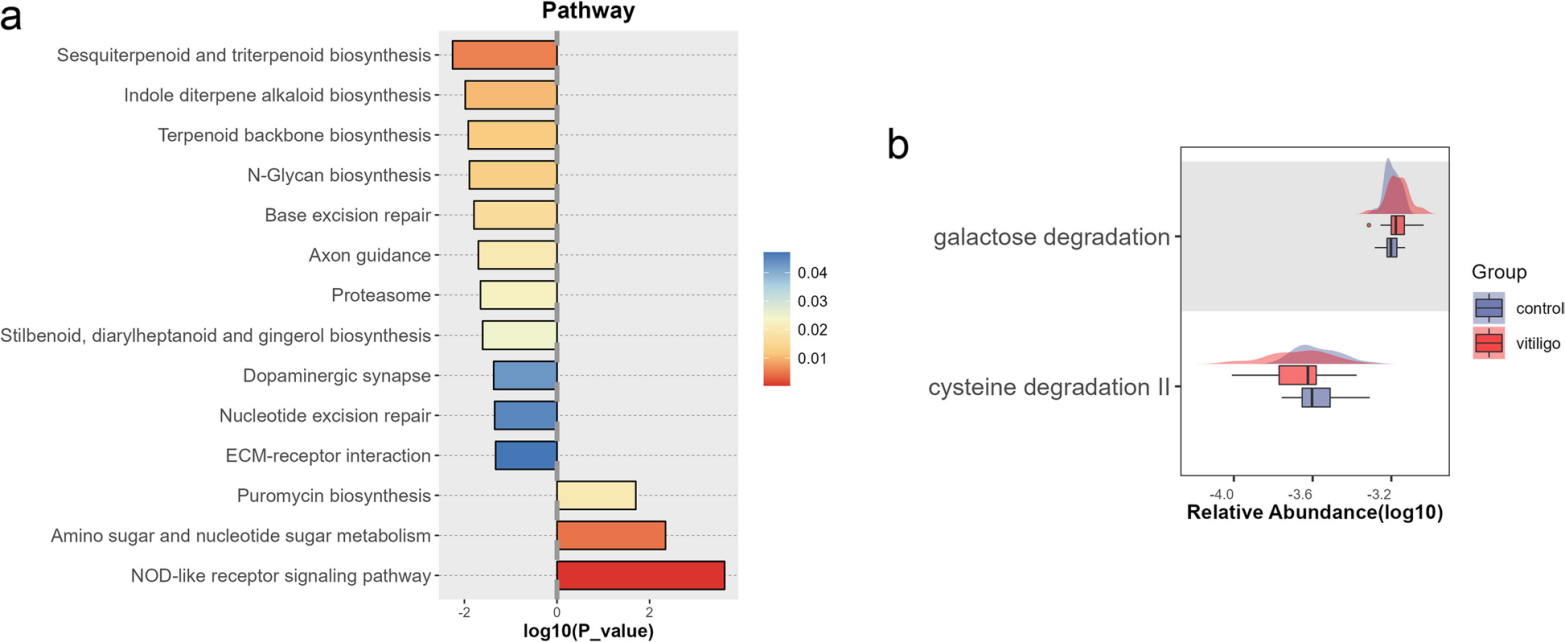
Analysis of gene function and metabolic pathways. **a** KEGG pathways demonstrated significant differences between vitiligo patients and HCs. **b** Galactose degradation and cysteine degradation II were identified as differential metabolic pathways

### Gene functions and metabolic pathways were differentially enriched between groups

Functional analysis revealed three major pathways that were enriched in vitiligo patients: amino sugar and nucleotide sugar metabolism, puromycin biosynthesis and NOD-like receptor signaling pathway (Fig. 4a; Supplementary Table S6). The most abundant pathways in the HCs were sesquiterpenoid and triterpenoid biosynthesis, indole diterpene alkaloid biosynthesis, terpenoid backbone biosynthesis, and N-glycan biosynthesis.

We constructed boxplots to represent the differential potential functions between vitiligo patients and HCs using GMMs, a method for analyzing the gut microbiota metabolism. The GMMs were extracted from the MetaCyc and KEGG databases and show the catabolism of carbohydrates, amino acids and lipids by prokaryotic anaerobes, cross-feeding interactions and production of fermentation end-products. GMMs analysis showed increased galactose degradation and decreased cysteine degradation II in patients with vitiligo (Fig. 4b; Supplementary Table S7).

**Fig. 5:**
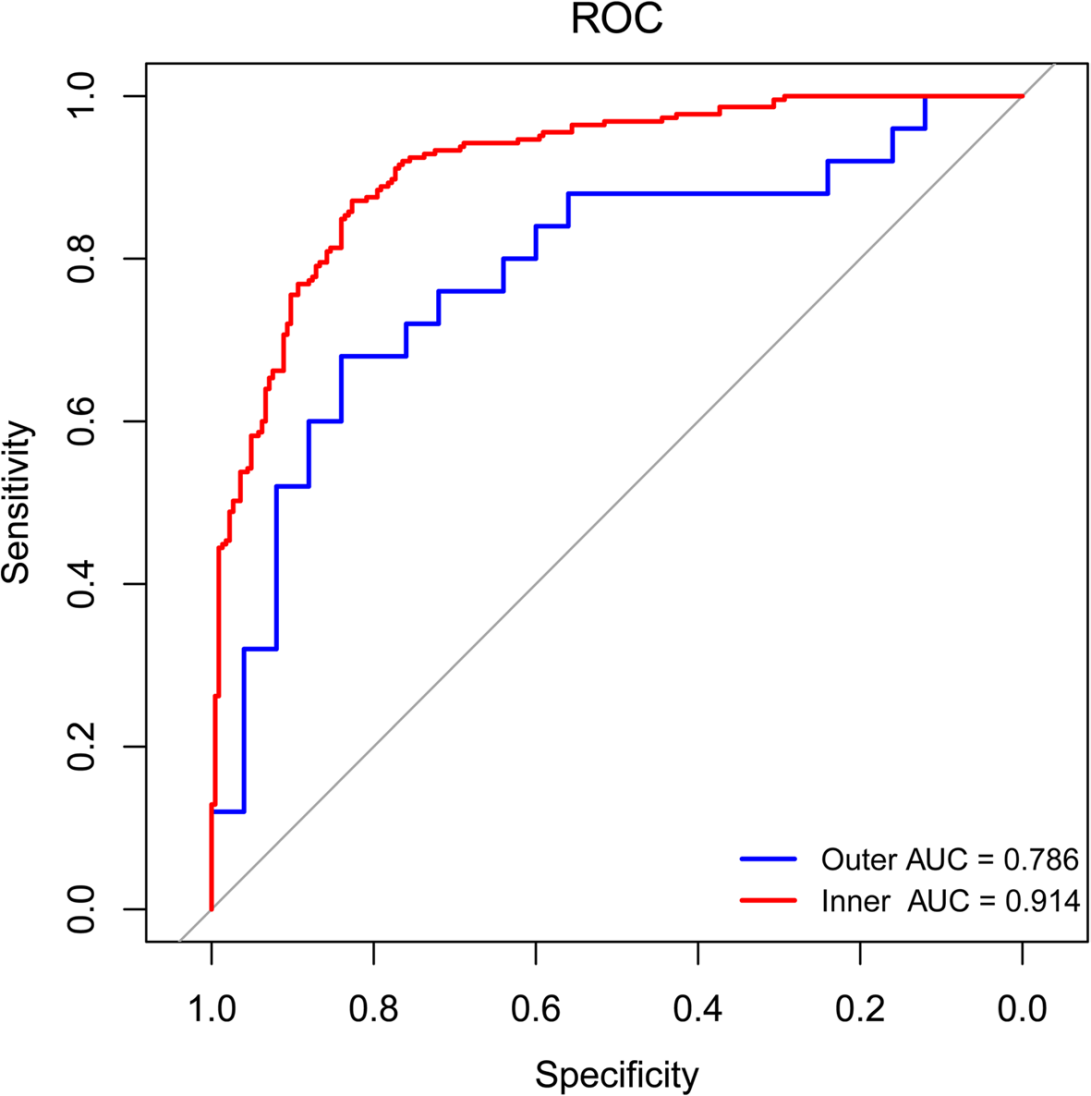
Diagnostic model based on 28 gut microbial species. Outer and inner ROC curve was calculated using a nest cross-validation model. Area under the ROC curve are shown

### Diagnostic model based on microbial species

Nested Cross-Validation model were constructed based on gut bacterial species to identify the gut bacteria related to vitiligo and evaluate their diagnostic values. We constructed a panel of 28 microbial features which include *Bacteroides fragilis*, *Lachnospiraceae bacterium BX3*, *TM7_phylum_sp_oral_taxon_348*, *Allisonella histaminiformans*, *Coprobacter_secundus*, *Lachnospiraceae_bacterium_OF09_6* ect (Supplementary Table S8). The area under the curve (AUC) for using the 28 gut microbes as markers to discriminate vitiligo patients from HCs was 0.786 (Fig. 5).

## Discussion

The concept of gut-skin axis has attracted much attention as more and more evidences suggest a link between dysregulation of gut microbiota and vitiligo. Compared with previous studies based on 16s rRNA sequencing, our study was the first to use metagenomic sequencing analysis to describe the species-level gut microbial compositions in patients with advanced vitiligo and revealed the gene functions and potential metabolic pathways of the gut microbes.

Our study revealed significantly reduced α-diversity in vitiligo patients compared with that of HCs (Fig. 1); this was consistent with the findings of Hanene et al. [14], but contradictory to the results of Ni et al. [15]. Lower α-diversity in the gut microbiota has also been reported in patients with psoriasis [12], alopecia areata [19] and Crohn's disease [20]. In our study, the most dominant bacterial phyla in the guts of vitiligo patients and control were *Bacillota, Bacteroidota, Actinomycetota* (Fig. 2a, b). *Bacillota* and *Bacteroidota* constituted 92.49% of the total microbial population in the patients, which were consistent with those of the HCs [21]. Compared with the previous results of 16s rRNA sequencing in patients with vitiligo, our study showed that the relative abundance of *Bacillota* was the highest, followed by *Bacteroidota*, which was consistent with the results of Hanene et al. [14]. Results of Ni et al. [15] showed that *Bacteroidota* had the highest abundance, followed by *Bacillota*. Our results showed that the ratio of *Bacillota/Bacteroidota* had no difference between the two groups, while the results of Hanene et al. [14] showed that the ratio increased in the disease group. The relative abundances of approximately 20 intestinal microbial species were lower in patients with vitiligo than in HCs (Fig. 2d). Intestinal bacteria that can produce short-chain fatty acids such as butyrate, including *Clostridium* spp, *Coprococcus comes* and *Streptococcus thermophilus* are reduced in patients with vitiligo [22]. In addition, butyrate, as a source of carbon, can enter the citric acid cycle and directly reduce the production of mitochondrial reactive oxygen species [23]. Oxidative stress plays significant roles in production of auto-antigens and subsequent autoimmunity in vitiligo [24–26]. We speculate that the relative deficiency of these gut bacteria leads to high oxidative stress throughout the bodies of vitiligo patients, which may promote the pathological process of vitiligo. *Streptococcus thermophilus* is a lactobacillus widely used to ferment yogurt [27]. Regulation of intestinal flora by oral probiotics can indirectly affect skin diseases, such as atopic dermatitis [28], and probiotics play a beneficial immune-modulatory role by reducing inflammation in patients with psoriasis [29]. Because *Streptococcus thermophiles* was decreased in patients with vitiligo, whether probiotics supplementation can be used as an optional adjuvant therapy for vitiligo needs to be validated by subsequent techniques.

LEfSe analysis (Fig. 3) showed that the most crucial bacterial species in vitiligo was *Bacteroides fragilis*, a normal component of the human gut microbiota. It has been reported that *Bacteroides fragilis*, as a symbiotic bacteria in the intestinal tract, can activate host immune response depending on the expression of the factor polysaccharide (PSA) [30]; PSA derived from *Bacteroides fragilis* could promotes human Treg function [31]. The abnormal increase of *Bacteroides fragilis* in vitiligo patients reflects the activation of immune response in pathological state, and cytokines produced by over-activated immune response may accelerate the pathological process of autoimmunity. *Lachnospiraceae bacterium BX3* is another bacterium abundant in vitiligo patients. Yilmaz et al. found that *Lachnospiraceae* were correlated with disease relapse after surgical interventions in patients with Crohn's disease [32]. Disturbances of *Lachnospiraceae* in inflammatory bowel disease and vitiligo reveal a connection between the gut and skin. The precise mechanisms behind these observations remain unclear.

We constructed a panel of 28 microbial features using a random forest model to distinguish vitiligo patients from HCs (Fig. 5). The identified bacteria specific to vitiligo coincided with the LEfSe analysis, *Bacteroides fragilis*, *Lachnospiraceae bacterium BX3*, *TM7_phylum_sp_oral_taxon_348* were enriched in vitiligo patients and *Allisonella histaminiformans*, *Coprobacter_secundus*, *Prevotella_copri_clade_C* were enriched in HCs. We believe that these species may be used to diagnose vitiligo and may have value in follow-up animal experiments in vivo (microbiota transplantation) to study the vitiligo pathogenesis.

The significant advantage of metagenomic sequencing over 16s rRNA sequencing is the analysis of intestinal bacterial gene functions. KEGG function analysis showed that the NOD-like receptor signaling pathway was enriched in patients with vitiligo (Fig. 4a). Nuclear localized leucine-rich repeat protein 1 (NLRP1), a member of the NOD-like receptor family, is considered to be an innate immune-modulator associated with vitiligo [33, 34]. NOD-like receptor thermal protein domain associated protein 3 (NLRP3) inflammasome can regulate keratinocyte innate immunity. Oxidative stress-induced keratinocyte derived NLRP3 activation promotes cutaneous T cell response and can be targeted to treat vitiligo [35]. Nucleotide oligomeric domain 2 (NOD2) receptors are classic pattern recognition receptors that are widely distributed in intestinal cells and can sense specific microbial peptidoglycan fragments, including muramyl dipeptide (MDP). *Firmicutes* phylum-derived DL-endopeptidase can activate the NOD2 signaling pathway by increasing the level of NOD2 ligand [36]. We believe that the pathogenesis of vitiligo autoimmunity may be caused by the following reasons: 1) It is possible that the upregulation of NOD-like receptor signaling pathway is due to auto-immunity causing self-antigen. In the pathological condition of vitiligo, the NOD signaling pathway is activated by the hydrolyzed fragments of intestinal microorganisms, thus causing inflammatory response, and these inflammatory cytokines accelerate the process of autoimmunity. 2) Some intestinal microorganisms can be sequentially homologous with melanocyte antigens, and as the orthologs of autoantigens, these bacterial fragments can induce auto-immunity. The bacterial species that can activate the NOD pathway in intestinal flora are widespread, but the potential specific bacterial species await further study.

GMMs analysis (Fig. 4b) showed that galactose degradation was enhanced in vitiligo. Galactose could induce oxidative stress, and galactose fermentation from the Leloir or tagatose pathways leads to formation of pyruvate, which can be converted to acetyl-CoA by pyruvate formate lyase [37]. Formation of acetyl-CoA can potentially produce additional oxygen radicals. *Bacteroide*s species are prevalent in the microbiome and are generally thought to be partly responsible for polysaccharide degradation [38]. A metabolic pathway utilizing L-galactose has been reported in *Bacteroides vulgatus* [39]. We speculated that *Bacteroide*s could affect the oxidative stress level of the body by affecting galactose metabolism and thus participate in the pathological process of vitiligo.

In this study, GMMs analysis revealed that cysteine degradation was reduced in vitiligo. Cysteine can be synthesized from homocysteine in the body. Previous studies reported that homocysteine metabolism is involved with the incidence and progression of vitiligo. Serum homocysteine levels are elevated in patients with vitiligo, and homocysteine can increase oxidative stress and inhibit the melanin synthesis enzyme, tyrosinase [40, 41]. Microbes in the gastrointestinal tract contribute to Hydrogen Sulfide (H_2_S) production in humans. *Desulfovibrio* is reported to be present in the gut and capable of produce H_2_S via Cysteine degradation [42]. Our results showed that the abundance of *Desulfovibrio* was significantly reduced in vitiligo compared with control (Fig. 2d), we hypothesize that hypercysteinemia may be caused by metabolic disorders in vitiligo patients, especially with reduced cysteine degradation ability, which is closely related to the deficiency of *Desulfovibrio* in vitiligo patients.

Our study had limitations. The sample size of the experiment is small and larger cohort and validation studies are needed to verify the identified microbial.

## Conclusions

In summary, we are the first to use metagenomic sequencing to compare the intestinal flora composition and its corresponding genetic and metabolic characteristics in patients with vitiligo and HCs. The intestinal microbial biomarkers we detected may be useful for early diagnosis and treatment of vitiligo. Future studies should use a multi-omics strategy to reveal microbial-derived metabolites involved in the pathological process of vitiligo. In vivo animal model experiments should be conducted to investigate associations between microorganisms and disease phenotypes.

### Methods

#### Participants

A total of 25 patients with advanced non-segmental vitiligo were recruited from the Department of Dermatology and Venereology, the First Affiliated Hospital of Xi'an Jiaotong University from May 2022 to August 2022. Twenty-five HCs matched for sex, age, and body mass index were also enrolled. All participants lived in Xi'an, Shaanxi Province, China. Exclusion criteria were the presence of other autoimmune or infectious diseases, or previous treatment with systemic antibiotics, or other immunosuppressive agents within the preceding three months. The Ethics Committee of the First Affiliated Hospital of Xi'an Jiaotong University approved the study. All participants have signed informed consent.

### Sample collection

Fecal samples were extracted using a rapid extraction kit (MGIEasy Stool Sample Collection Kit; Shanghai, China) and promptly stored in a -80°C refrigerator after sampling.

### Metagenomic sequencing

DNA was extracted from the stool samples using the Magbeads fast DNA^®^ kit (MP Biomedicals, Shanghai, China) following the manufacturer's instructions. The concentrations of the extracted DNA were measured by NanoDrop 2000, followed by 1% agarose gel electrophoresis for integrity detection Using KAPA HyperPlus PCR-free reagent (Illumina, CA, US), DNA was trimmed to an average of approximately 400 bp to construct a paired-end library. The details are as follows: 500 ng of nucleic acid was interrupted to a main band ≈ 420 bp with Covaris, and the main band with 380–420 bp were screened. 50 ng of fragment-screened DNA was used for library construction, including procedures of end repair, A-tailing addition, sequencing adapters linking, purification, PCR amplification, and PCR product purification, etc.. Qubit 2.0 (Thermo Fisher, Massachusetts, US) was used to accurately quantify the library, and a total amount of library greater than 300ng was needed. Quality control on the distribution of library fragments was performed with Agilent 2100 (Agilent, Palo Alto, US) to ensure the main peak to be around 500bp. Then, pooling was performed on all sample libraries and the total amount of DNA after pooling was required to be 300 ng, and the volume is ≤ 48ul. Following heat denaturation, single-strand circularization, enzyme digestion and purification, Shotgun metagenomic sequencing was performed on BGI platform (MGESEQ-T7, Shenzhen, China) by Shaan Probiomicros (Xi’an, China). Low-quality reads and adaptors were dumped from the raw reads by fastp (v0.23.0) (length_required = 50, n_base_limit = 2), and the host reads were excluded based on the human genomes 38 using Bowtie2 software (v2.3.5.1). Finally, clean reads averaging 15.05 Gb and 15.23 Gb of high-quality sequences were acquired per sample from the patients and HCs, respectively.

### Taxonomic profiling

After quality control of the sequencing data, MetaPhlAn (v3.0.5) [43] was used to identify the microbiota and predict the relative abundances at different taxonomic levels. Microbiota richness and distributions were estimated by α-diversity using the Shannon and Simpson indexes. Compositional differences between the vitiligo patients and HCs (β-diversity) were measured via PCoA according to the Bray-curtis distance. Permutational multivariate analysis of variance (PERMANOVA) was used to test the influence of group and other factors on gut microbiota. Wilcoxon rank sum test was used to calculate the abundance differences between groups. LEfSe [44] analysis was applied to screen the specific intestinal microbial taxa that could represent each group.

### Functional analysis

The GMMs were used to analyze microbial functional changes [45]. GMM reflect bacterial and archaeal metabolism unique to the human intestinal environment, with emphasis on anaerobic fermentation processes. Briefly, 103 GMM were identified using Bowtie2(v2.3.5.1) [46, 47], which was built through extensive literature review and metabolic database, including KEGG [48] and MetaCyc. MetaCyc hierarchy of pathway classifications were used to reconstruct the metagenome of each sample into metabolic pathways based on annotations.

Using BLASTP (v2.12.0) of each gene (http://gigadb.org/dataset/100253) for the KO allocation, each protein through comments hit with the highest scores assigned to KO, which contains at least one high score clips to score more than 60. Pathway results were obtained by mapping relationship between KO and pathway. KO matrix were obtained by comparing our metagenomic data to the KO, and then through mapping relationship between GMM and KO, we obtain a matrix of GMM. The differences in the enrichment of microbial gene functions based on the KEGG database and GMM between groups were analyzed.

### Nested Cross-Validation model

Three hundred thirty-four microbial species were selected with a non-zero value rate > 10%. R package nested CV (version V0.6.1) was used to construct receiver operating characteristic (ROC) curve of this mode. The number of outer folds and inner folds were set to 10. Filter functions (random forest variable importance) for feature selection were selected, and it can be embedded within the outer loop of the nested cross validation (CV). Finally, 28 bacteria were selected and ROC curves from left-out folds from both outer and inner CV were plotted. The AUC based on the left-out outer folds is the unbiased estimate of accuracy, while the left-out inner folds demonstrate bias due to the optimisation of the model’s hyperparameters on the inner fold data.

### Statistical analysis

All data were analyzed using R software (v4.0.2). Fisher's exact test was used to analyze the unordered classification variables which are reported as counts and proportions. Wilcoxon test (corrected by the Benjamini-Hochberg method, false discovery rate < 0.05) was used to detect the relative abundance differences between groups and KO. LEfSe analysis with LDA values > 2.0 and all *P* < 0.05 were considered statistically significant. Spearman rank correlation was used to analyze the correlation between the differential microbial abundance and VIDA score in patients with vitiligo.

### Supplementary Information

**Additional file 1: Supplementary Table 1.** Summary of statistics of α-diversity. **Supplementary Table 2.**  Summary of statistics of β-diversity. **Supplementary Table 3.**  Summary of statistics of different gut microbiome taxonomy between control and vitiligo. **Supplementary Table 4.**  The correlation between different microbial species and VIDA score. **Supplementary Table 5.** Analysis of bacterial taxa by using LEfSe analyses. **Supplementary Table 6.**  Analysis of KEGG pathway. **Supplementary Table 7.**   Analysis of metabolic pathways. **Supplementary Table 8.**  Microbial species selected based on nest cross-validation model. 

## Data Availability

Metagenomic raw sequencing data for biosamples have been deposited in SRA database with BioProject ID PRJNA1013076. Raw data can be accessed here: https://www.ncbi.nlm.nih.gov/bioproject/PRJNA1013076

## Abbreviations

HCs: Healthy Controls
KEGG: Kyoto Encyclopedia of Gene and Genomes
VIDA: Vitiligo Disease Activity
PCoA: Principal Coordinate Analysis
PERMANOVA: Permutational Multivariate Analysis of Variance
LDA: Linear Discriminant Analysis
LEfSe: Linear Discriminant Analysis Effect Size
GMMs: Gut Metabolic Modules
AUC: Area Under the Curve
NLRP1: Nuclear Localization Leucine-rich-repeat Protein 1
NLRP3: Nuclear Localization Leucine-rich-repeat Protein 3
NOD2: Nucleotide oligomeric domain 2
KO: KEGG orthologys
ROC: Receiver Operating Characteristic
CV: cross validation
